# PDGFRβ is an essential therapeutic target for BRCA1-deficient mammary tumors

**DOI:** 10.1186/s13058-021-01387-x

**Published:** 2021-01-21

**Authors:** Feng Bai, Shiqin Liu, Xiong Liu, Daniel P. Hollern, Alexandria Scott, Chuying Wang, Lihan Zhang, Cheng Fan, Li Fu, Charles M. Perou, Wei-Guo Zhu, Xin-Hai Pei

**Affiliations:** 1 Guangdong Provincial Key Laboratory of Regional Immunity and Diseases, International Cancer Center, Shenzhen University Health Science Center, Shenzhen, 518060 China; 2 Department of Pathology, Shenzhen University Health Science Center, Shenzhen, 518060 China; 3 Dewitt Daughtry Family Department of Surgery, University of Miami, Miami, FL 33136 USA; 4 Department of General Surgery, Xiangya Hospital, Central South University, Changsha, China; 5 Department of Anatomy and Histology, Shenzhen University Health Science Center, Shenzhen, 518060 China; 6 Lineberger Comprehensive Cancer Center, University of North Carolina at Chapel Hill, Chapel Hill, NC 27599 USA; 7 The Second Affiliated Hospital of Xi’an Jiaotong University, Xi’an, 710061 China; 8 The Affiliated Cancer Hospital of Zhengzhou University, Zhengzhou, 450008 China; 9 Department of Pharmacology, Shenzhen University Health Science Center, Shenzhen, 518039 China; 10 Department of Biochemistry and Molecular Biology, International Cancer Center, Shenzhen University Health Science Center, Shenzhen, 518060 China

**Keywords:** BRCA1, PDGFRβ, EMT, Mammary tumor, Targeted therapy

## Abstract

**Background:**

Basal-like breast cancers (BLBCs) are a leading cause of cancer death due to their capacity to metastasize and lack of effective therapies. More than half of BLBCs have a dysfunctional BRCA1. Although most *BRCA1*-deficient cancers respond to DNA-damaging agents, resistance and tumor recurrence remain a challenge to survival outcomes for BLBC patients. Additional therapies targeting the pathways aberrantly activated by BRCA1 deficiency are urgently needed.

**Methods:**

Most BRCA1-deficient BLBCs carry a dysfunctional INK4-RB pathway. Thus, we created genetically engineered mice with Brca1 loss and deletion of p16^*INK4A*^, or separately p18^I*NK4C*^, to model the deficient INK4-RB signaling in human BLBC. By using these mutant mice and human *BRCA1-*deficient and proficient breast cancer tissues and cells, we tested if there exists a druggable target in BRCA1-deficient breast cancers.

**Results:**

Heterozygous germline or epithelium-specific deletion of *Brca1* in p18^I*NK4C*^- or p16^*INK4A*^-deficient mice activated Pdgfrβ signaling, induced epithelial-to-mesenchymal transition, and led to BLBCs. Confirming this role, targeted deletion of Pdgfrβ in *Brca1*-deficient tumor cells promoted cell death, induced mesenchymal-to-epithelial transition, and suppressed tumorigenesis. Importantly, we also found that pharmaceutical inhibition of Pdgfrβ and its downstream target Pkcα suppressed *Brca1*-deficient tumor initiation and progression and effectively killed *BRCA1*-deficient cancer cells.

**Conclusions:**

Our work offers the first genetic and biochemical evidence that PDGFRβ-PKCα signaling is repressed by BRCA1, which establishes PDGFRβ-PKCα signaling as a therapeutic target for *BRCA1*-deficient breast cancers.

**Supplementary Information:**

The online version contains supplementary material available at 10.1186/s13058-021-01387-x.

## Background

The heterogeneity of breast cancer is marked by pathologically distinct tumor types that differ in their responsiveness to treatment. In the clinical setting, breast cancer is comprised of three main subtypes: HER2-positive, estrogen receptor (ER)-positive luminal, and basal-like cancers (BLBCs) that make up the majority of triple-negative breast cancer [TNBC; ER, PR, HER2 negative by immunohistochemistry (IHC) analysis] [1, 2]. BLBCs are highly heterogeneous and aggressive, perhaps due to their enrichment of tumor-initiating cells (TICs) or cancer stem cells that are thought to drive clinical relapse and metastasis [3–6]. TICs and tumor cells with mesenchymal features have enhanced capacity to metastasize and are resistant to radio- and chemotherapy [7]. Mammary TICs can be generated from luminal tumor cells by an epithelial-to-mesenchymal transition (EMT) program [7–15], a process in which epithelial cells lose many of their epithelial characteristics and acquire mesenchymal features [7]. BLBCs likely originate from luminal progenitors [8–11] and contain a number of distinct cell types including cells that express luminal biomarkers [16–18]. Notably, more than half of BLBCs are associated with functional loss of BRCA1, caused by germline or somatic mutation or by promoter hypermethylation [19–22]. BRCA1 is a tumor suppressor that functions in DNA damage repair. Although the majority of BRCA1-deficient cancer patients respond to DNA-damaging agents such as cisplatin and poly (ADP-ribose) polymerase (PARP) inhibitors, tumor recurrence and resistance, likely driven by TICs, combine to decrease the 5-year survival of such patients [23, 24]. Thus, additional therapies targeting the pathways aberrantly activated by BRCA1 deficiency are urgently needed.

In addition to BRCA1 loss, many of the DNA-repair-deficient TNBCs also harbor a dysfunctional INK4-RB pathway [21, 25–27]. Key participants in this pathway are p16^*INK4A*^ (p16) and p18^I*NK4C*^ (p18). These two members of the inhibitors of the CDK4/6 (INK4) family inhibit CDK4 and CDK6, whose activation phosphorylates and functionally inactivates the RB family of proteins (RB, p107, p130) [26]. Importantly, p16 is inactivated in ~ 30–60% of breast cancers, and p18 expression is reduced in these cancers {[21, 28–30] and Bai unpublished data}. We and others have reported that BRCA1 deficiency in human and mouse mammary epithelial cells (MECs) activates the p16 and p18, inducing premature senescence [11, 31–34]. Finally, we have demonstrated that loss of *p16* or *p18* rescues the premature senescence of MECs caused by *Brca1* deficiency [11, 31, 33, 34] and that loss of *Brca1* in *p16-* or *p18-*deficient mice leads to the development of mammary tumors resembling BLBC accompanied by features of EMT [11, 31].

PDGFRβ, a receptor for members of the platelet-derived growth factors (PDGFs), is abundantly expressed in stromal fibroblasts [35–37]. PDGFRβ signaling plays a critical role in activating cancer-associated fibroblasts (CAFs) which facilitate breast cancer growth and progression [35, 38, 39]. Notably, PDGFRβ expression is specifically upregulated in late-stage breast cancer cells [40–42]. PDGFRβ activates PKCα which then phosphorylates FRA1, a key EMT-inducing transcription factor (EMT-TF), and drives TIC function of transformed MECs [43, 44]. Inhibition of PDGFRβ-PKCα signaling specifically targets oncogene-transformed MECs that have undergone EMT and induces mesenchymal-to-epithelial transition (MET), suppressing tumor initiation and progression [40, 44, 45]. It remains elusive how PDGFRβ signaling is involved in epithelial tumorigenesis. For example, whether PDGFRβ signaling activates EMT in a tumor cell autonomous manner and whether PDGFRβ-PKCα signaling is required for survival and for maintaining mesenchymal status of the tumor cells in vivo are key unanswered questions that may inform treatment strategies for breast cancer.

In this study, we examined the molecular outcomes of BRCA1 deficiency combined with loss of either p16 or p18 to identify targetable pathways in BRCA1/INK4-RB-deficient tumors. This identified activation of the Pdgfrβ pathway as potential driver in these tumors. Further, we showed preclinical evidence that these molecular alterations in Pdgfrβ pathway could be effectively therapeutically targeted.

## Methods

### Mice, histopathology, and immunostaining

*Brca1*^*f/f*^ and Tg (MMTV-Cre) 4Mam mice were obtained from the NCI Mouse Repository and JAX lab, respectively [46, 47]. The generation of *p16*^*−/−*^, *p18*^*−/−*^, *Brca1*^*+/−*^, *Brca1*^*MGKO*^ *(Brca1*^*f/f*^;MMTV-Cre or *Brca1*^*f/−*^;MMTV-Cre) mice was described previously [31, 48–50]. The Institutional Animal Care and Use Committee at the University of Miami and Shenzhen University approved all animal procedures. Histopathology and immunohistochemistry (IHC) were performed as described previously [11, 28, 31]. The primary antibodies used were Ck14, cleaved caspase 3 (Thermal Scientific), PDGFRβ, phosphorylated PKCα (p-PKCα), phosphorylated FRA1 (p-FRA1), FRA1 (Cell signaling), ERα, BRCA1 (Santa Cruz), E-cadherin (E-cad), PKCα (BD Biosciences), and Vimentin (Vim) (Abcam). Immunocomplexes were detected by using the Vectastain ABC DAB kit according to the manufacturer’s instructions (Vector Laboratories) or by using FITC- or rhodamine-conjugated secondary antibodies (Jackson Immunoresearch).

### Mammary tumor cell preparation and tumorsphere formation assay

Mammary tumors were dissected and tumor cell suspensions were prepared as previously described [11, 28, 31]. For the primary and secondary tumorsphere formation assays, primary *p18*^*−/−*^*;Brca1*^*MGKO*^ mammary tumor cells were plated onto six-well, ultra-low attachment plates, in serum-free DMEM-F12 supplemented with B27, EGF, and bFGF as described [28, 31]. Primary tumorspheres formed were collected and dissociated after 9 days of culture. 10^4^ cells dissociated from the primary tumorsphere were plated in triplicate with or without treatment. Secondary tumorspheres that formed after 6 days of culture were counted under the microscope.

### Cell culture, cell viability assay, overexpression of BRCA1, CRISPR-mediated Pdgfrβ knockout, drug treatment, and annexin V analysis

T47D, HCC1937, MCF7, SUM149, MDA-MB231, and BT20 cells were cultured per ATCC recommendations. To determine cell viability, 50,000 cells were plated in 24-well plates and treated with DMSO, PDGFR tyrosine kinase inhibitor III (PDGFR inh III, EMD Biosciences) [44, 51], or RO31-8220, a specific PKCα inhibitor (Cayman Chemical) [44, 52], at the indicated concentrations. Viable cell numbers were determined by an automatic cell counter (Bio-rad). Dead cells were determined by trypan blue or propidium iodide (PI) staining, and the percentage of dead cells was calculated from at least 1000 cells. For ectopic expression of BRCA1, cells were transfected with pBabe-empty, pBabe-HA-BRCA1, or pBabe-Myc-BRCA1 as previously described [31]. For CRISPR-mediated Pdgfrβ knockout (KO) in primary tumor cells, Pdgfrβ CRISPR/Cas9 KO plasmids (mouse) (Santa Cruz, SC-422171) were transfected into *p18*^*−/−*^*;Brca1*^*MGKO*^ primary tumor cells following the manufacturer’s protocol. GFP-positive and GFP-negative cells were sorted 2 days after transfection on a BD FACS SORP Aria-IIu machine for further analysis. For annexin V analysis, *p18*^*−/−*^*;Brca1*^*MGKO*^ tumor cells were transfected with the Pdgfrβ CRISPR/Cas9 KO plasmids, and 48 h after transfection, cells were stained with annexin V-pacific blue (Biolegend) and analyzed by the LSR–Fortessa machine (BD Pharmingen). Data analysis was performed using Kaluza software (Beckman Coulter).

### Transplantation and tumor treatment

For in vivo transplantation, *p18*^*−/−*^*;Brca1*^*MGKO*^ primary tumor cells transfected with the Pdgfrβ CRISPR/Cas9 KO plasmids were FACS sorted. Six thousand GFP^neg^ and GFP^pos^ live cells (trypan blue negative) were suspended in a 50% solution of Matrigel (BD) and then inoculated into the left and right inguinal mammary fat pads (MFPs) of 6-week-old female NSG mice (Jackson Laboratory), respectively. Four weeks after transplantation, animals were euthanized and mammary tumors were dissected for histopathological and immunohistochemical analyses. For ex vivo transplantation, primary *p18*^−/−^;*Brca1*^MGKO^ tumor cells were cultured to generate primary tumorsphere. 10^4^ cells dissociated from primary tumorspheres were treated with DMSO, Ro-31-8220, or PDGFR inh III for 6 days. One thousand live cells were transplanted into MFPs of NSG mice. Four weeks after transplantation, animals were euthanized and mammary tumors were analyzed. For treatment of established mammary tumors, *p18*^−/−^;*Brca1*^MGKO^ tumor cells were transplanted into MFPs of NSG mice and allowed to reach ~ 200 mm^3^ in size. Mice were then treated with daily i.p. injection of the agents, and the tumor size was determined.

### Microarray analysis, western blot, and ChIP assay

RNA was extracted and purified from tumors using an RNeasy kit (Qiagen). Tumor RNA was reverse transcribed, amplified, and labeled with Cy5, and WT mammary tissue reference RNA was reverse transcribed, amplified, and labeled with Cy3. The amplified sample and reference were co-hybridized to Agilent 4x180k custom mouse microarrays and were analyzed as described previously [14, 53]. Murine tumor gene expression data was deposited at the Gene Expression Omnibus under accession number GSE155239. Single sample gene set enrichment analysis (ssGSEA) was performed using GenePattern [54]. The gene signature for PDGF signaling was taken from Reactome [55], while EMT and mammary stem cell signatures were published [56, 57].

For the western blot, tissue and cell lysates were prepared as previously reported [28, 31]. The primary antibodies used were BRCA1 (Santa Cruz), E-cad, Vim, PDGFRβ, p-PDGFRβ, p-PKCα, p-Fra1 and Snail (Cell signaling), Gapdh (Ambion), and Twist (Abcam). ChIP assays were carried out as previously described [28, 31]. Briefly, T47D and HCC1937 cells were treated with 1.5% formaldehyde and sonicated. Anti-BRCA1 antibody (D-9, Santa Cruz) or control mouse IgG was used to precipitate chromatin associated with BRCA1. Q-PCR was performed to determine the relative abundance of target DNA. Specific primers for the analysis of BRCA1 binding to PDGFRβ are listed in Additional file 1.

### Human tumor samples and gene expression datasets

Formalin-fixed paraffin-embedded (FFPE) human breast cancer samples lacking patient-identifying information were obtained with IRB approval from the Tissue Banks at the University of Miami and the Department of Pathology at Shenzhen University. All samples obtained were non-treated invasive breast cancers with known ER status. The MetaBric human breast cancer dataset [58] was analyzed for correlation between BRCA1 with PDGFRβ and PKCα mRNA levels.

### Statistical analysis

All data are presented as the mean ± SD for at least three repeated individual experiments for each group. Quantitative results were analyzed by the two-tailed Fisher exact test or two-tailed Student’s *t* test. *p* < 0.05 was considered statistically significant.

## Results

### Heterozygous germline deletion of *Brca1* in *p18*-deficient mice leads to basal-like tumors with activation of EMT and increase of Pdgfrβ

**Fig. 1:**
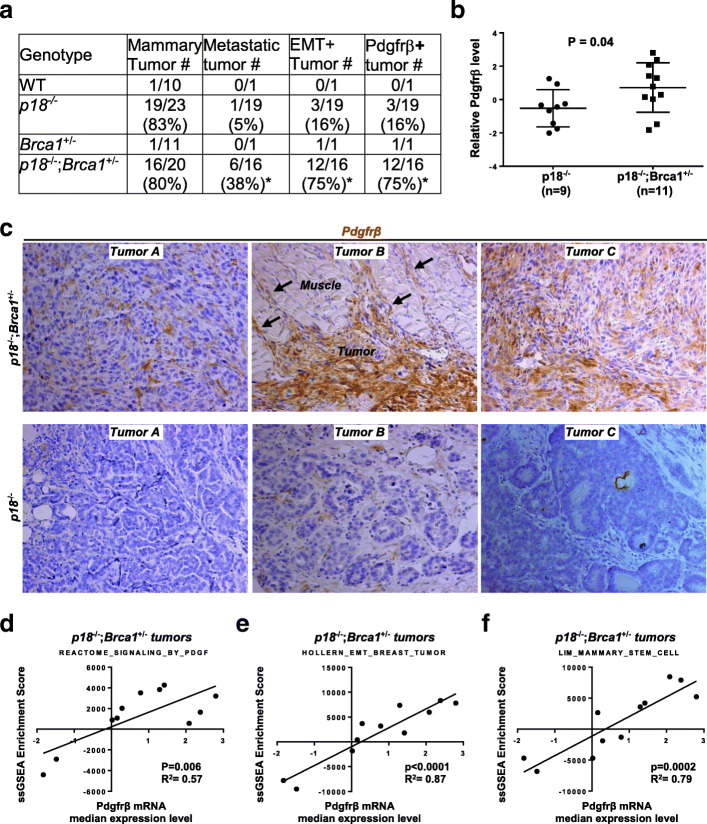
Heterozygous germline deletion of Brca1 in p18-deficient mice activates EMT with increase of Pdgfrβ in mammary tumors. **a** Summary of mammary tumors in mice with Balb/c background. Metastasis was found in the lung. EMT + tumors are defined as tumors that exhibit two of the following: decreased E-Cad, increased Vim, Fn1, or Cd29, and are positive for two EMT-transcription factors (Twist, Snail, Slug, Foxc2, or Fra1) in > 2% of cells. Pdgfrβ+ tumors are tumors that are positive for Pdgrfβ in > 2% tumor cells. The asterisk (*) denotes a significance from *p18*^*−/−*^*;Brca1*^*+/−*^ and *p18*^*−/−*^ tumors by a two-tailed Fisher’s exact test. **b** Microarray analysis of tumors. Boxplot showing relative Pdgfrβ mRNA levels in *p18*^*−/−*^*;Brca1*^*+/−*^ (*n* = 11) and *p18*^*−/−*^ (*n* = 9) tumors. **c** Representative immunostaining of mammary tumors from a *p18*^−/−^ and two *p18*^−/−^;*Brca1*^+/−^ mice. Note that *p18*^*−/−*^*;Brca1*^*+/−*^ tumor cells that invade into surrounding muscle are strongly positive for Pdgfrβ (tumor B in top panel) and that most Pdgfrβ-positive cells in *p18*^−/−^ tumors are stromal cells (tumor B in bottom panel). Pdgfrβ-positive *p18*^*−/−*^*;Brca1*^*+/−*^ tumor cells at the tumor invasion front are indicated. **d**–**f** *X*-*Y* plot showing the relation of Pdgfrβ mRNA and ssGSEA enrichment scores for signatures of Pdgf signaling activity [55] (**d**), EMT in breast cancers [56] (**e**), and mammary stem cells [57] (**f**)

We previously discovered that the majority of *p18*^−/−^ mice developed CK8^+^ and ER^+^ luminal type mammary tumors while most *p18*^−/−^;*Brca1*^+/−^ mice formed mammary tumors that were ER^−^ and CK5^+^ basal-like tumors expressing mesenchymal markers as well as EMT-TFs [11, 28, 31]. In addition, *p18*^−/−^;*Brca1*^+/−^ mammary tumors were enriched with cancer stem cells [31, 59] and significantly more metastatic than *p18*^−/−^ tumors (Fig. 1a). These data suggest that heterozygous germline deletion of *Brca1* induces basal-like tumors with activation of EMT and promotes metastasis. To further explore the role of PDGFRβ signaling in activating EMT and promoting tumor initiation and progression [40, 44, 45], we performed a microarray analysis of mammary tumors from *p18*^−/−^ and *p18*^−/−^;*Brca1*^+/−^ mutant mice. Analysis of differentially expressed genes identified Pdgfrβ mRNA to be significantly higher in p18^−/−^;Brca1^+/−^ tumors than in p18^−/−^ tumors (Fig. 1b). We then performed IHC analysis and detected that 75% (*n* = 16) of *p18*^−/−^;*Brca1*^+/−^ tumors expressed Pdgfrβ, whereas only 16% (*n* = 19) of *p18*^−/−^ tumors yielded similar results. Strong Pdgfrβ expression ranged from 2 to 60% of *p18*^−/−^;*Brca1*^+/−^ tumor cells, while Pdgfrβ expression was found in 2–5% of *p18*^−/−^ tumor cells and was much weaker in intensity. All the EMT-positive *p18*^−/−^;*Brca1*^+/−^ tumors were also positive for Pdgfrβ (Fig. 1a, c). We also noted *p18*^−/−^;*Brca1*^+/−^ tumor cells that had invaded into muscles were marked by high levels of Pdgfrβ expression (Fig. 1c). Taking advantage of the majority of *p18*^−/−^;*Brca1*^+/−^ tumors expressing a high level of Pdgfrβ and a few of these tumors expressing a low level of Pdgfrβ, we performed ssGSEA and found that the lowest Pdgfrβ samples had lower Pdgf signaling as expected (Fig. 1d, Additional file 2A). Furthermore, Pdgfrβ mRNA levels in *p18*^−/−^;*Brca1*^+/−^ mammary tumors strongly correlated with EMT and stem cell signatures (Fig. 1e, f, and Additional file 2B, C, D), in agreement with the data derived from IHC and published elsewhere [31, 59]. Together, these results suggest that germline deletion of Brca1 in a *p18*^−/−^ background activates EMT in mammary tumorigenesis, which is associated with an increase of Pdgfrβ and Pdgfrβ-activated signaling pathway.

### Specific deletion of *Brca1* in *p18* and *p16* null epithelium activates Pdgfrβ-Pkcα signaling and EMT inducing metastatic basal-like tumors

Pdgfrβ is abundantly expressed in stromal fibroblasts which also play an important role in facilitating breast cancer growth and progression [35, 38, 39]. To confirm the role of Brca1 loss in regulating Pdgfrβ in mammary epithelial and carcinoma cells, and to rule out the effect of stromal Pdgfrβ regulated by Brca1 loss in mammary tumorigenesis, we generated *Brca1*^*MGKO*^ *(Brca1*^*f/f*^;MMTV-Cre or *Brca1*^*f/−*^;MMTV-Cre), *p18*^*−/−*^*;Brca1*^*MGKO*^, and *p16*^*−/−*^*;Brca1*^*MGKO*^ mice in the Balb/c-B6 mixed background in which Brca1 was specifically deleted in mammary epithelia. We previously reported the successful deletion of Brca1 and the activation of EMT in mammary epithelia of *p18*^*−/−*^*;Brca1*^*MGKO*^ and *p16*^*−/−*^*;Brca1*^*MGKO*^ mice [31]. We found that 47% (*n* = 15) of *p18*^−/−^, 73% (*n* = 15) of *p18*^*−/−*^*;Brca1*^*MGKO*^, and 60% (*n* = 10) of *p16*^*−/−*^*;Brca1*^*MGKO*^ mice in the Balb/c-B6 mixed background developed mammary tumors, whereas only 8% (*n* = 13) of *Brca1*^*MGKO*^ and no *p16*^*−/−*^ mice did so at similar ages (Table 1). Notably, 36% (*n* = 11) of *p18*^*−/−*^*;Brca1*^*MGKO*^ and 50% (*n* = 6) of *p16*^*−/−*^*;Brca1*^*MGKO*^ tumors, but no *p18*^−/−^ tumors, metastasized to the lung (Table 1), consolidating the role of loss-of-function of Brca1 in promoting tumor metastasis.

**Table 1:** Specific deletion of *Brca1* induces mammary tumors with activation of Pdgfrβ signaling in *p18-* and *p16-*deficient mice

Genotype^1^	Mammary tumor #	Metastatic tumor #^3^	EMT+ tumor #^4^	Pdgfrβ+ tumor #^5^	p-Pkcα+ tumor #^6^	p-Fra1+ tumor #^6^
WT	0/9					
*p18*^*−/−*^	7/15 (47%)	0/7	2/7 (29%)	2/7 (29%)	2/7 (29%)	2/7 (29%)
*Brca1*^*MGKO2*^	1/13 (8%)	0/1	1/1	1/1	1/1	1/1
*p18*^*-/-*^;*Brca1*^*MGKO*^	11/15 (73%)	4/11 (36%)	9/11 (82%)*	9/11 (82%)*	9/11 (82%)*	9/11 (82%)*
*p16*^*-/-*^	0/20					
*p16*^*-/-*^;*Brca1*^*MGKO*^	6/10 (60%)	3/6 (50%)	6/6 (100%)	6/6 (100%)	6/6 (100%)	6/6 (100%)

^1^All mice were in Balb/c-B6 mixed background

^2^*Brca1*^*MGKO*^, *Brca1*^*f/f*^;MMTV-Cre, or *Brca1*^*f/-*^;MMTV-Cre

^3^Metastasis was found in the lung

^4^At least two EMT markers (decreased E-Cad, increased Vim, Fn1, Sma or Cd29) or two EMT-TFs, which included Twist, Slug, Snail, Foxc1, and Foxc2, were detected in > 2% tumor cells by IHC

^5^Tumors that had > 2% positive Pdgfrβ cells by IHC, or primary *p18*^*-/-*^*;Brca1*^*MGKO*^ and *p16*^*-/-*^*;Brca1*^*MGKO*^ tumor cells at passage 1 expressed fivefolds more Pdgfrβ than *p18*^*-/-*^ tumor cells by western blot analysis (see Fig. 2e)

^6^Tumors that had > 2% positive p-Pkcα or p-Fra1 cells by IHC

*A significance from *p18*^*-/-*^*;Brca1*^*MGKO*^ and *p18*^*-/-*^ tumors by a two-tailed Fisher’s exact test

**Fig. 2:**
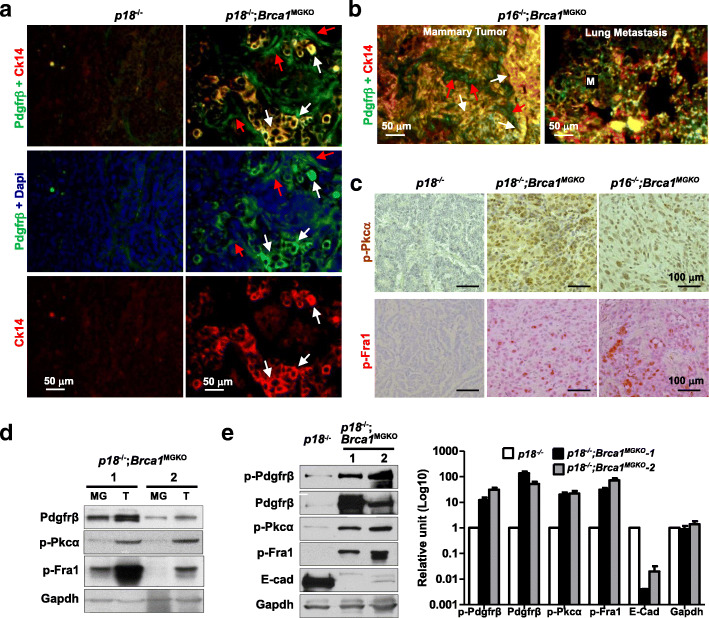
Deletion of Brca1 in *p18*^−/−^ or *p16*^−/−^ epithelia activates Pdgfrβ-Pkcα signaling and EMT in basal-like tumors. **a**–**c** Representative immunostaining of tumors from *p18*^−/−^, *p18*^−/−^;*Brca1*^MGKO^, and *p16*^−/−^;*Brca1*^MGKO^ mice. Pdgfrβ^+^ tumor cells (Ck14^+^ Pdgfrβ^+^, white arrows) and stromal cells (Pdgfrβ^+^, red arrow) are indicated. Note the widely spread Pdgfrβ and Ck14 doubly positive cells in a *p16*^−/−^;*Brca1*^MGKO^ primary mammary tumor and its lung metastasis (**b**). **d** Tumors (T) from two independent *p18*^−/−^;*Brca1*^MGKO^ mice (mouse 1, 10 months old; and mouse 2, 18 months old) were analyzed by western blot. Tumor-free mammary glands (MG) from the same mouse were used as controls. **e** Tumor cells from three independent mice were cultured and analyzed. Protein bands were quantified by Image-Pro Plus 6.0

Though the mammary tumor incidence of *p18*^−/−^ mice in the Balb/c-B6 background was lower than that of the Balb/c background, *p18*^−/−^ tumors in the Balb/c-B6 background were also predominantly luminal (Fig. 1a and Table 1) [28, 31]. Eighty-two percent (*n* = 11) of *p18*^*−/−*^*;Brca1*^*MGKO*^ and all of *p16*^*−/−*^*;Brca1*^*MGKO*^ tumors were positive for Vim, EMT-TFs, and Pdgfrβ, whereas only 29% (*n* = 7) of *p18*^−/−^ tumors were positive for these features. Further, comparing these *p18*^*−/−*^*;Brca1*^*MGKO*^ and *p16*^*−/−*^*;Brca1*^*MGKO*^ tumors with p18^−/−^ tumors, we identified more tumors with high p-Pkcα and p-Fra1 which together indicated Pdgfrβ pathway activity in Brca1-deficient tumors (Table 1, Fig. 2a–c).

Implying epithelial origin, most Pdgfrβ-positive *p18*^*−/−*^*;Brca1*^*MGKO*^ and *p16*^*−/−*^*;Brca1*^*MGKO*^ mammary tumor cells were also positive for Ck14, an epithelial marker. Notably, Pdgfrβ and Ck14 doubly positive tumor cells were also detected in lung metastases of *p18*^*−/−*^*;Brca1*^*MGKO*^ and *p16*^*−/−*^*;Brca1*^*MGKO*^ mammary tumors (Fig. 2a, b, and Additional file 3), which demonstrated that the metastases were derived from Brca1-deficient basal-like mammary tumors with an increased Pdgfrβ expression. Using western blots to assess protein, we found that the expression of Pdgfrβ, p-Pkcα, and p-Fra1 was clearly increased in *p18*^−/−^;*Brca1*^*MGKO*^ tumors relative to that of adjacent tumor-free mammary tissues in the same mice or to *p18*^−/−^ tumors (Fig. 2d, Additional file 4), and in *p18*^−/−^;*Brca1*^*MGKO*^ tumor cells relative to that in *p18*^−/−^ tumor cells (Fig. 2e). As a whole, these data suggest that loss of Brca1 induces EMT and metastatic basal-like tumors in an epithelium-autonomous manner and that Brca1 loss activates Pdgfrβ-Pkcα signaling in epithelial tumor cells thereby enriching the number of tumor cells with EMT-like features.

### BRCA1 inhibits PDGFRβ-PKCα signaling and EMT

**Fig. 3:**
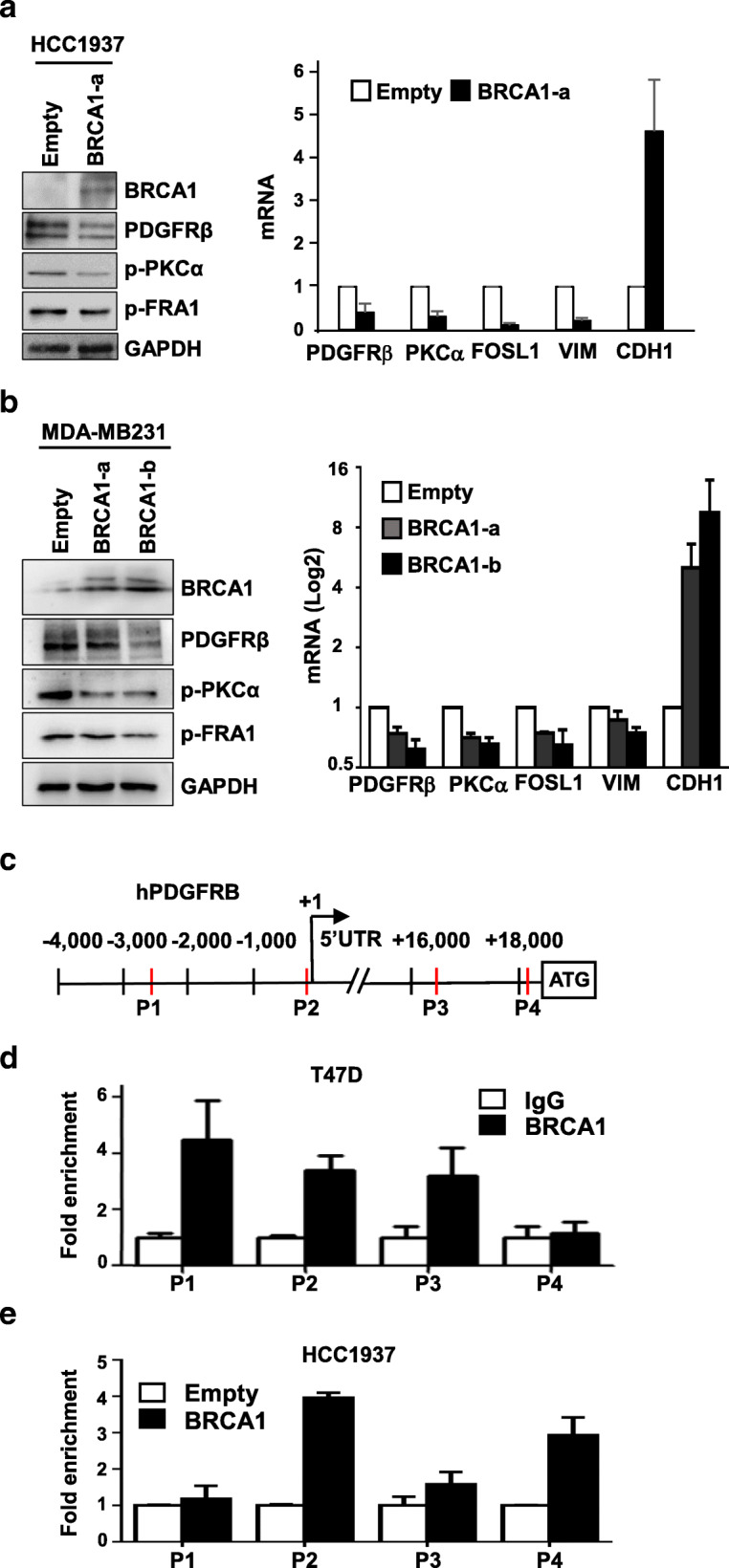
BRCA1 binds to PDGFRβ locus and represses transcription of PDGFRβ and EMT-associated genes. **a**, **b** HCC1937 (**a**) and MDA-MB231 (**b**) cells were transfected with pBabe-empty (Empty), pBabe-HA-BRCA1 (BRCA1-a), or pBabe-Myc-BRCA1 (BRCA1-b). Expression of genes indicated in the cells were determined by western blot and qRT-PCR 48 h after transfection. **c** Diagram showing the location of putative BRCA1 binding sites (red bars) in the human PDGFRβ gene. + 1, transcription start site. **d** ChIP analysis of endogenous BRCA1 binding to the PDGFRβ locus in T47D cells. The results were normalized to the amount of input and compared with the IgG-negative controls. Data are represented as mean ± SD. **e** ChIP analysis of exogenous BRCA1 binding to the PDGFRβ locus in HCC1937 cells transfected with Babe-empty (Empty) or pBabe-HA-BRCA1 (BRCA1). The results were normalized to the amount of Input and compared with the empty controls. Data are represented as mean ± SD

To confirm the role of BRCA1 in controlling Pdgfrβ-Pkcα signaling, we overexpressed WT BRCA1 in two BRCA1-mutant breast cancer cell lines, HCC1937 and SUM149, and a basal-like cell line, MDA-MB231. BRCA1 inhibited the mRNA and protein levels of PDGFRβ indicating BRCA1 repressed transcription of PDGFRβ (Fig. 3a, b, and Additional file 5). Consistently, the level of phosphorylation of PKCα was also reduced in BRCA1-overexressed cells confirming that BRCA1 suppressed PDGFRβ-PKCα signaling (Fig. 3a, b, and Additional file 5). Furthermore, we found that overexpression of BRCA1 in these cells induced expression of CDH1 (encoding E-cadherin) while inhibiting expression of FOSL1 (encoding FRA1) and VIM, consistent with our finding that deficiency of Brca1 induced EMT in tumor cells. Importantly, these results confirm the role of Brca1 in suppressing EMT in a mammary tumor cell autonomous manner.

### BRCA1 binds to the PDGFRβ locus to repress its transcription

We and others have shown that BRCA1 binds to FOXC1/2 and TWIST loci to repress their transcription [31, 60]*.* Our bioinformatic analysis revealed that there exist at least four putative BRCA1 binding sites in the PDGFRβ locus (Fig. 3c). We performed chromatin-immunoprecipitation (ChIP) assays and found that endogenous and exogenous BRCA1 bound to the PDGFRβ locus in T47D and HCC1937 cells, respectively (Fig. 3d, e). Together with the inhibitory effect of BRCA1 for the expression of PDGFRβ, these data suggest that BRCA1 binds to the PDGFRβ locus to repress its transcription.

### Targeted deletion of Pdgfrβ in Brca1-deficient tumor cells promotes cell death and MET suppressing tumorigenesis

**Fig. 4:**
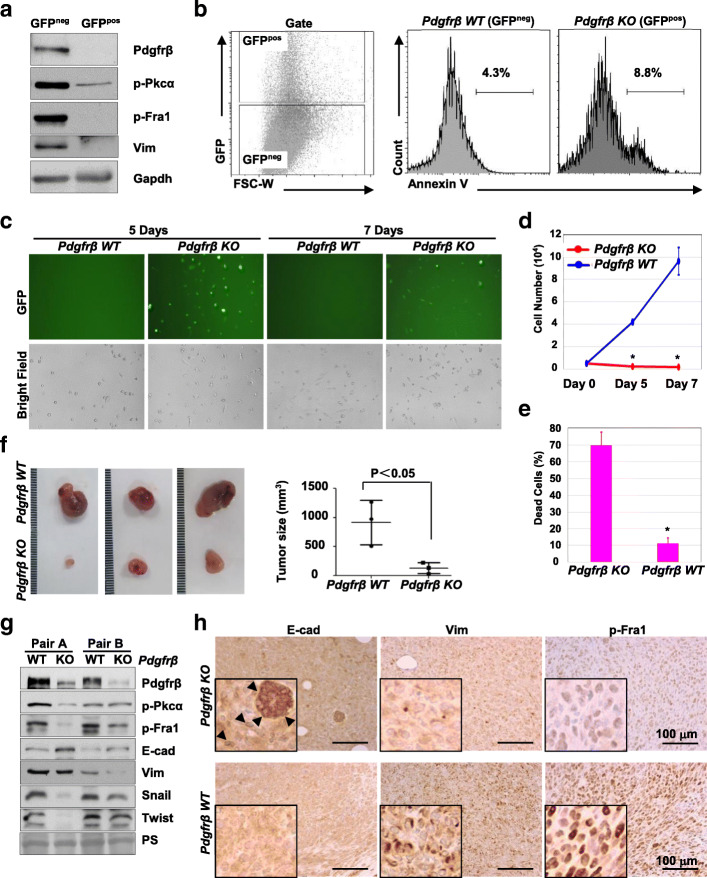
Deletion of Pdgfrβ in Brca1-deficient tumor cells promotes cell death, induces MET, and suppresses tumorigenesis. **a** Primary *p18*^−/−^;*Brca1*^MGKO^ tumor cells were transfected with Pdgfrβ CRISPR/Cas9 KO plasmids. Forty-eight hours later, GFP^neg^ and GFP^pos^ cells were sorted out and analyzed by western blot. **b** *p18*^−/−^;*Brca1*^MGKO^ cells were transfected with Pdgfrβ CRISPR/Cas9 KO plasmids. After 48 h, annexin V-positive rate in Pdgfrβ WT (GFP^neg^) and Pdgfrβ KO (GFP^pos^) cells was determined by flow cytometry. **c**–**e** Primary *p18*^−/−^;*Brca1*^MGKO^ tumor cells were transfected with Pdgfrβ CRISPR/Cas9 KO plasmids. Forty-eight hours later, FACS-sorted Pdgfrβ WT (GFP^neg^) and Pdgfrβ KO (GFP^pos^) cells were cultured and monitored for additional 7 days (**c**). The number of viable cells at day 5 and day 7 was counted (**d**), and the percentage of dead cells at day 7 were calculated (**e**). The asterisk (*) denotes a significance from Pdgfrβ WT and Pdgfrβ KO cells at day 5 or day 7. **f** Freshly sorted Pdgfrβ WT and KO *p18*^−/−^;*Brca1*^MGKO^ tumor cells that were viable were transplanted into MFP of NSG mice. Four weeks later, regenerated tumor volumes were determined. Data are represented as mean ± SD of three tumors in each group. **g**, **h** Tumors generated from **f** were analyzed by western blot (**g**) and IHC (**h**). PS, ponceau staining. The insets show the enlarged cells that are representative. E-cad-positive cells are indicated by arrowheads

To determine the role of Pdgrfβ in Brca1-deficient tumorigenesis, we knocked out Pdgfrβ in *p18*^*−/−*^*;Brca1*^*MGKO*^ primary tumor cells by using the CRISPR/Cas9 system. We transfected Pdgfrβ CRISPR/Cas9 KO plasmids (encoding GFP) into *p18*^*−/−*^*;Brca1*^*MGKO*^ primary tumor cells. Two days after transfection, GFP-negative (GFP^neg^) and GFP-positive (GFP^pos^) cells were FACS sorted for further analysis. We found that GFP^pos^ tumor cells expressed nearly no detectable Pdgfrβ and drastically reduced p-Pkcα, p-Fra1, and Vim, when compared with GFP^neg^ tumor cells (Fig. 4a). These data confirmed successful depletion of Pdgfrβ protein in the GFP^pos^ *p18*^*−/−*^*;Brca1*^*MGKO*^ mammary tumor cells. Additionally, these data also indicate that activation of Pkcα and its downstream target Fra1 is dependent on Pdgfrβ signaling, and Pdgfrβ signaling is required for maintaining the mesenchymal traits in Brca1-deficient tumor cells.

To test the impact of Pdgfrβ CRISPR/Cas9 KO on tumor cell viability, we performed annexin V staining. We found that 8.8% of GFP^pos^ (Pdgfrβ KO) tumor cells were positive for annexin V compared to 4.3% of the GFP^neg^ (Pdgfrβ WT) cells (Fig. 4b). These data indicate that acute deletion of Pdgfrβ slightly enhances apoptosis of Brca1-deficient tumor cells. We further analyzed FACS-sorted Pdgfrβ WT and Pdgfrβ KO *p18*^*−/−*^*;Brca1*^*MGKO*^ tumor cells in dishes and observed that the population with characteristic morphological features of apoptosis (i.e., cell shrinkage, pyknosis, dense cytoplasm with tightly packed organelles) or dead cells was significantly increased in Pdgfrβ KO cells relative to that in Pdgfrβ WT cells after 5–7 days of culture (Fig. 4c). We found that the viable cells were significantly less and the dead cells were drastically more in Pdgfrβ KO population than in Pdgfrβ WT population (Fig. 4d, e). These data indicate that long-term depletion of Pdgfrβ in *p18*^*−/−*^*;Brca1*^*MGKO*^ tumor cells in vitro increases tumor cell death.

We next transplanted freshly FACS-sorted Pdgfrβ WT and Pdgfrβ KO *p18*^*−/−*^*;Brca1*^*MGKO*^ tumor cells that were viable into MFPs of NSG mice. We found that Pdgfrβ WT *p18*^*−/−*^*;Brca1*^*MGKO*^ tumor cells generated palpable tumors in 10–14 days whereas Pdgfrβ KO cells did not generate palpable tumors in the same time period. Four weeks after transplantation, tumors generated by Pdgfrβ KO cells were significantly smaller than tumors generated by Pdgfrβ WT cells (Fig. 4f). We performed western blot in conjunction with IHC analysis and observed that tumors generated by Pdgfrβ KO cells expressed clearly reduced levels of Pdgfrβ, p-Pkcα, p-Fra1, Vim, Snail, and Twist, but increased levels of E-cad, when compared with tumors generated by Pdgfrβ WT cells (Fig. 4g, h, and data not shown). Notably, we detected that some cells in tumors generated by Pdgfrβ KO *p18*^*−/−*^*;Brca1*^*MGKO*^ cells expressed high levels of E-cad, whereas E-cad-positive cells in tumors generated by Pdgfrβ WT *p18*^*−/−*^*;Brca1*^*MGKO*^ tumor cells were rarely observed (Fig. 4h). These results demonstrate that deletion of Pdgfrβ in Brca1-deficient tumor cells promotes MET and suppresses tumorigenesis.

### Inhibition of Pdgfrβ or Pkcα activity suppresses Brca1-deficient tumor initiating potential

**Fig. 5:**
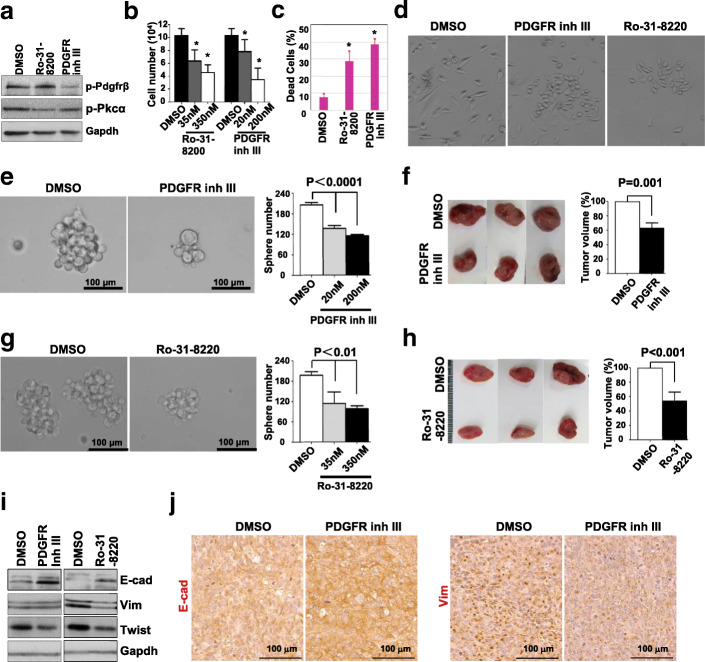
Inhibition of Pdgfrβ and Pkcα activity promotes MET and suppresses Brca1-deficient tumor initiation. **a** *p18*^−/−^;*Brca1*^MGKO^ tumor cells treated with PDGFR Inh III at 20 nM or Ro-31-8220 at 35 nM for 30 min were analyzed by western blot. **b**–**d** *p18*^−/−^;*Brca1*^MGKO^ tumor cells were treated with PDGFR Inh III or Ro-31-8220 for 3 days. The number of viable cells was determined (**b**). The percentage of dead cells treated with PDGFR Inh III at 200 nM or Ro-31-8220 at 350 nM (**c**) and the morphology of cells treated with PDGFR Inh III at 20 nM or Ro-31-8220 at 35 nM (**d**) were also analyzed. Data in **b** and **c** are represented as the mean ± SD of triplicate experiments. The asterisk (*) denotes a significance from DMSO- and drug-treated cells. **e**, **g** Primary *p18*^−/−^;*Brca1*^MGKO^ tumor cells were cultured to generate primary tumorsphere. 10^4^ cells dissociated from primary *p18*^−/−^;*Brca1*^MGKO^ tumorspheres were treated with PDGFR Inh III at 20 nM and 200 nM (**e**) or Ro-31-8220 at 35 nM and 350 nM (**g**). Secondary tumorspheres formed after 6-day treatment were counted from triplicate experiments. Data are represented as the mean ± SD. **f**, **h** Cells dissociated from primary *p18*^−/−^;*Brca1*^MGKO^ tumorspheres were treated with PDGFR Inh III at 20 nM or Ro-31-8220 at 35 nM. Six days after treatment, 1000 viable cells pretreated with DMSO, PDGFR Inh III (**f**), or Ro-31-8220 (**h**) were transplanted into MFP of NSG mice. Four weeks later, tumor volumes were determined. Data are represented as mean ± SD of four tumors in each group. **i**, **j** Tumors generated in **f** and **h** were analyzed by western blot (**i**) and IHC (**j**)

To determine whether Pdgfrβ and Pkcα activity represented therapeutic target in BRCA1-deficient tumors, we treated *p18*^*−/−*^*;Brca1*^*MGKO*^ tumor cells with a PDGFR Inh III and PKCα inhibitor, Ro-31-8220. The activity and specificity of these drugs in inhibiting phosphorylation of PDGFRβ and PKCα have been well confirmed by multiple groups [44, 51, 52, 61]. Illustrating these inhibitors were having the intended effects, we observed reduced Pdgfrβ and Pkcα phosphorylation following treatment (Fig. 5a, and Additional file 6A, B). In addition, we examined the impact of dosage on tumor cells. Treatment of *p18*^*−/−*^*;Brca1*^*MGKO*^ tumor cells with PDGFR Inh III and Ro-31-8220 significantly reduced cell number and increased cell death, particularly with high dosages (200 nM for PDGFR Inh III and 350 nM for Ro-31-8220) (Fig. 5b, c). At a low dosage (20 nM for PDGFR Inh III and 35 nM for Ro-31-8220), these drugs converted spindle-shaped, mesenchymal-like cells into epithelial-like cells (Fig. 5d). This is consistent with the data derived from targeted deletion of Pdgfrβ, and these results further confirm that inhibition of Pdgfrβ-Pkcα signaling promotes Brca1-deficient tumor cell death and MET.

We have previously reported that deletion of Brca1 in p18-deficient mice activates EMT and enhances TICs [31]. We detected that *p18*^*−/−*^*;Brca1*^*MGKO*^ tumor cells formed significantly more and larger spheres than *p18*^*−/−*^ cells (data not shown) confirming the role of loss-of-function of Brca1 in stimulating TICs. We treated *p18*^*−/−*^*;Brca1*^*MGKO*^ tumorsphere-dissociated cells with PDGFR Inh III or Ro-31-8220 and found that inhibition of Pdgfrβ or Pkcα activity significantly reduced secondary tumorsphere forming potential (Fig. 5e, g). Moreover, we transplanted *p18*^*−/−*^*;Brca1*^*MGKO*^ tumorsphere-dissociated cells into NSG mice and found that cells pretreated with low-dose PDGFR Inh III or Ro-31-8220 produced significantly smaller tumors than control cells (Fig. 5f, h). Western blot and IHC analysis revealed that tumors generated by PDGFR Inh III or Ro-31-8220 pretreated cells expressed higher levels of E-cad and lower levels of Vim (Fig. 5i, j) than DMSO-pretreated cells. Again, these results confirm that the inhibition of Pdgfrβ and Pkcα activity promotes MET and reduces Brca1-deficient tumor initiating potential.

### Inhibition of Pdgfrβ or Pkcα activity suppresses established Brca1-deficient tumor progression

**Fig. 6:**
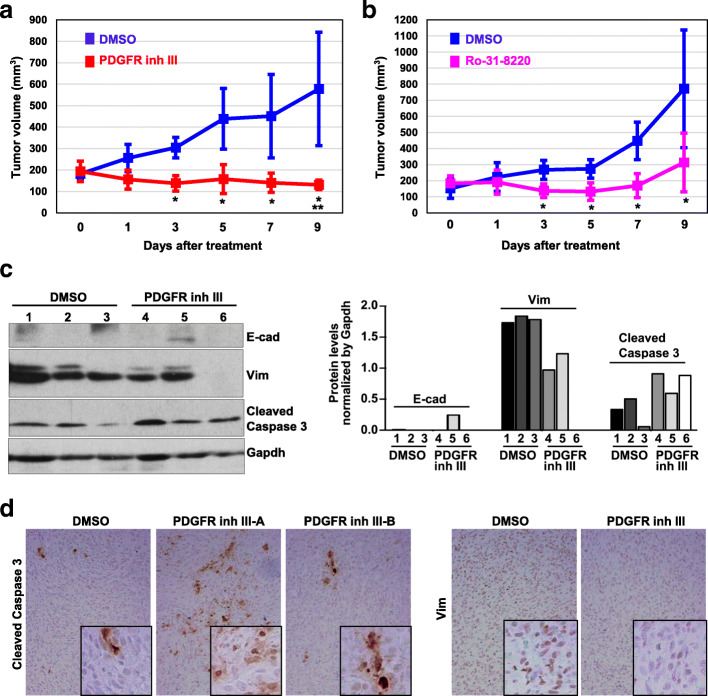
Inhibition of Pdgfrβ or Pkcα activity reduces EMT and promotes apoptosis suppressing established Brca1-deficient tumor progression. **a**, **b** *p18*^−/−^;*Brca1*^MGKO^ tumor cells were transplanted into MFPs of NSG mice and allowed to reach ~ 200 mm^3^ in size. Mice were then treated with daily i.p. injection of DMSO or PDGFR Inh III at 5 mg/kg (**a**) and DMSO or Ro-31-8220 at 5 mg/kg (**b**), respectively. The tumor size was determined and plotted. Data are represented as mean ± SD of four (**a**) and five (**b**) tumors in each group. *p < 0.05 between two groups at each time point by Student *t* test. **p < 0.05 between the group that was at the start of treatment and the group that was treated with PDGFR Inh III for 9 days. **c** Representative tumors treated with DMSO (three tumors) or PDGFR Inh III (three tumors) for 9 days were analyzed by western blot (left). The indicated protein levels of each lane were quantified and normalized by that of Gapdh (right). Note the increased cleaved caspase 3 and decreased Vim in PDGFR Inh III-treated samples (lanes 4, 5, and 6) in comparison with those in DMSO-treated samples (lanes 1, 2, and 3). **d** Representative tumors treated with DMSO or PDGFR Inh III for 9 days were analyzed by IHC. The insets show the enlarged cells that are representative

We then determined if pharmaceutical inhibition of Pdgfrβ and Pkcα activity had any effect on the progression of established Brca1-deficient tumors. Transplanted *p18*^*−/−*^*;Brca1*^*MGKO*^ tumors were allowed to reach 150–200 mm^3^ in size and then mice were treated with DMSO or inhibitors daily. Three days after treatment, tumors from PDGFR Inh III- or Ro-31-8220-treated mice began to show a significant size reduction in comparison with the tumors from DMSO-treated animals (Fig. 6a, b). After a 9-day treatment, tumors from DMSO-treated mice reached 578 ± 265 mm^3^ in size, whereas those from PDGFR Inh III-treated mice only reached 131 ± 22 mm^3^, which was even smaller than that of the tumor size at the start of treatment (194 ± 48 mm^3^) (Fig. 6a), indicating that treatment with PDGFR Inh III induced regression of Brca1-deficient tumors. Consistently, animals that received Ro-31-8220 treatment for 9 days had a significant reduction in tumor size compared with DMSO treatment controls (772 ± 366 mm^3^ vs. 313 ± 182 mm^3^, Fig. 6b). These data demonstrate that pharmaceutical inhibition of Pdgfrβ and Pkcα activity has therapeutic effects on established Brca1-deficient tumors.

To determine the mechanisms associated with inhibition of tumor progression by Pdgfrβ and Pkcα inhibitors, we analyzed Brca1-deficient tumors by western blot. This revealed that all tumors treated with PDGFR Inh III or Ro-31-8220 expressed more cleaved caspase 3 and less Vim (Fig. 6c, and data not shown), and one expressed higher levels of E-cad when compared with those with DMSO treatment (tumor 5 in Fig. 6c, and data not shown). IHC analysis confirmed that all tumors treated with PDGFR Inh III or Ro-31-8220 displayed more cleaved caspase 3-positive cells than control tumors, though the level of increase varied among tumors and different areas of an individual tumor. All control tumors widely expressed high levels of Vim while PDGFR Inh- or Ro-31-8220-treated tumors showed faint or undetectable Vim (Fig. 6d, and data not shown). In sum, these results suggest that inhibition of Pdgfrβ or Pkcα activity suppresses tumor progression by reducing mesenchymal features and inducing apoptosis.

### Inhibition of PDGFR or PKCα activity efficiently kills BRCA1-deficient human breast cancer cells

**Fig. 7:**
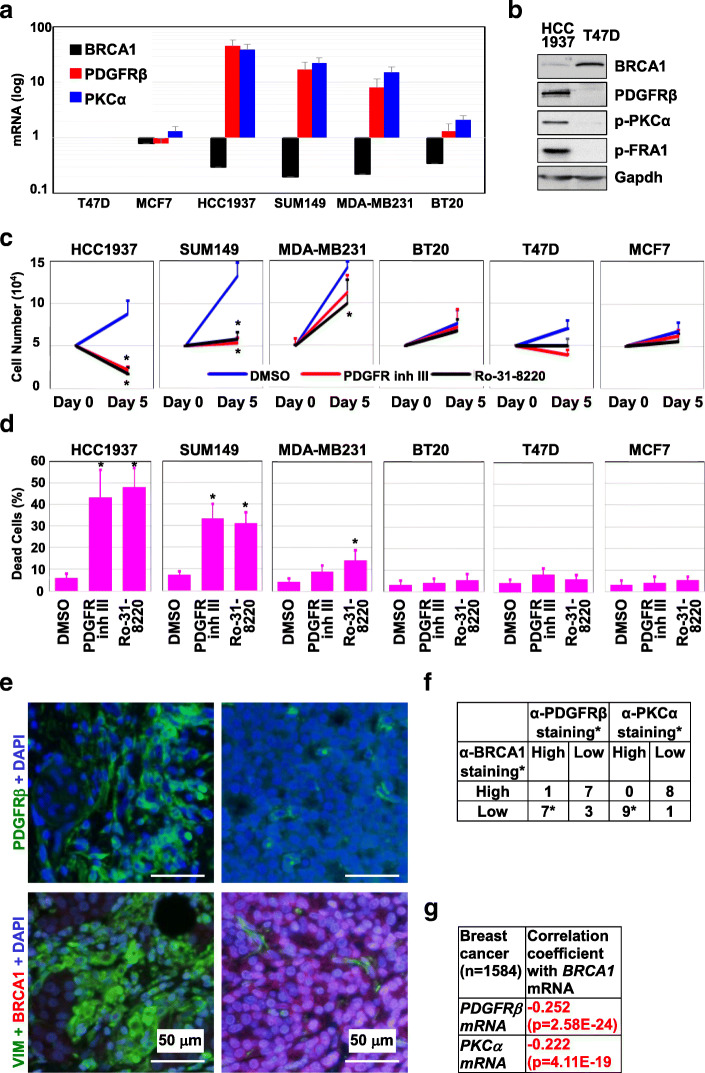
Inhibition of PDGFRβ or PKCα activity targets BRCA1-deficient breast cancer cells, and expression of BRCA1 is inversely related with that of PDGFRβ and PKCα in breast cancers. **a** Expression of BRCA1, PDGFRβ, and PKCα mRNA in human breast cancer cell lines was determined by qRT-PCR. **b** Expression of the proteins indicated in HCC1937 and T47D cells was determined by western blot. **c**, **d** Cancer cells were treated with DMSO, PDGFR Inh III (20 nM), or Ro-31-8220 (35 nM) and the number of viable cells (**c**) and dead cells (**d**) was determined after 5 days. Data are represented as mean ± SD of triplicates. The asterisk (*) denotes a significance from DMSO- and drug-treated cells. **e** Representative immunostaining analysis of serial human breast cancer sections. **f** Summary of the immunostaining results for human breast cancer samples. * “High” expression represents the samples positively stained with an antibody in more than 2% cells (i.e., scores equal to or higher than “+” in Additional file 7B). “Low” expression represents the samples negative or positively stained with an antibody in less than 2% cells (i.e., scores less than “+/−” in Additional file 7B). The asterisk (*) denotes a significance from BRCA1 high and BRCA1 low tumors by a two-tailed Fisher’s exact test. **g** Correlation analysis of mRNA levels of BRCA1 and PDGFRβ or PKCα for MetaBric breast cancer patients

To confirm the cytotoxic effects of inhibition of PDGFR and PKCα on BRCA1-deficient human breast cancer cells, we screened six widely used cell lines including two BRCA1 WT luminal lines, T47D and MCF7; two BRCA1 WT basal lines, MDA-MB231 and BT20; and two BRCA1 mutated lines, HCC1937 and SUM149. Importantly, all these cell lines carry defective INK4-RB pathway (undetectable p16 in T47D, MCF7, MDA-MB231, BT20, SUM149, and undetectable RB in HCC1937) [62–64], which are similar with our p18^−/−^, p18^−/−^;Brca1^MGKO^, and p16^−/−^;Brca1^MGKO^ mouse tumor cell system. We observed that the expression of PDGFRβ and PKCα was drastically increased in two BRCA1 mutant cell lines (HCC1937 and SUM149) and a BRCA1 lowly expressing MDA-MB231 line, whereas PDGFRβ and PKCα levels were extremely low in BRCA1 WT luminal lines (T47D and MCF7) and slightly increased in a BRCA1 WT basal line (BT20) (Fig. 7a, b). Western blot analysis revealed that BRCA1 mutant HCC1937 cells expressed significantly higher amounts of PDGFRβ, p-PKCα, and p-FRA1 than BRCA1-WT T47D cells (Fig. 7b) which suggested the activation of PDGFRβ-PKCα signaling by BRCA1 loss, consistent with our finding in mice and the findings from others [44, 65]. We confirmed that PDGFR inh III and Ro-31-8220 suppressed PDGFRβ and PKCα activities in HCC1937 and T47D cells, though the latter of which exhibited extremely low level of PDGFRβ and PKCα activities (Additional file 6A, B). We treated these cells with the inhibitors and found that PDGFR Inh III and Ro-31-8220 significantly reduced the number of viable cells and promoted cell death in BRCA1-mutated HCC1937 and SUM149 cells. In BRCA1 lowly expressing MDA-MB231 cells, these effects were moderate and even more slight in BRCA1 WT luminal cells (Fig. 7c, d, Additional file 6C). Importantly, BRCA1 in BT20 cells was lower than that in luminal cells (MCF7 and T47D), but not as low as that in MDA-MB231 cells. Thus, the slightly increased level of PDGFRβ and PKCα in BT20 cells relative to that in luminal cells suggested that other factors were involved in inactivation of PDGFRβ and PKCα and may explain the poor response to PDGFRβ and PKCα inhibitors in BT20 cells. Together, these data suggest that inhibition of PDGFR or PKCα activity efficiently kills BRCA1-deficient human breast cancer cells.

### Expression of BRCA1 is inversely related to that of PDGFRβ and PKCα in breast cancers

To extend these results to human breast tumors, we performed immunostaining analysis of 8 ER^+^ and 10 ER^−^ primary human breast cancers. We found that PDGFRβ, PKCα, FRA1, and VIM were readily detected in ER^−^ and BRCA1 weak or non-detectable tumor cells, whereas these proteins were barely detectable in ER^+^ and BRCA1^+^ tumor cells (Fig. 7e and Additional file 7A, B). Further analysis revealed that PDGFRβ and PKCα detected by immunostaining were significantly inversely correlated with BRCA1 expression (Fig. 7f). We then analyzed PDGFRβ and PKCα mRNA expression in TCGA breast cancer dataset and did not find significant differences of the expression between BRCA1 mutant and WT TNBCs (data not shown). This may be partly resulted from the small sample size of BRCA1 mutant TNBCs in TCGA dataset (PDGFRβ and PKCα mRNA data are available in only five BRCA1 mutant TNBCs). Moreover, we queried the mRNA expression of genes in the METABRIC 1584 breast cancer sample sets and found a significant inverse correlation between BRCA1 with PDGFRβ and PKCα mRNA levels (Fig. 7g). These clinical findings, consistent with our results in mice, further confirm that BRCA1 suppresses the PDGFRβ-PKCα signaling pathway in breast basal-like cancer development and progression.

## Discussion

In this study, we showed that heterozygous germline deletion of or epithelium-specific deletion of Brca1 in p18-deficient or p16-deficient mice led to basal-like tumors with elevated markers of EMT and Pdgfrβ-Pkcα signaling activity. We demonstrated that BRCA1 bound to the PDGFRβ locus, repressed PDGFRβ transcription, and inhibited EMT. Targeted deletion of Pdgfrβ in Brca1-deficient tumor cells inactivated Pdgfrβ-Pkcα signaling, promoted cell death, induced MET, and suppressed tumorigenesis. Consistently, pharmaceutical inhibition of Pdgfrβ or Pkcα activity suppressed Brca1-deficient tumor initiation and progression. We also found that inhibition of PDGFR or PKCα activity efficiently killed BRCA1-deficient human breast cancer cells and that expression of BRCA1 was inversely related to that of PDGFRβ and PKCα in human breast cancer samples. These data not only confirm that BRCA1 suppresses EMT and basal-like tumorigenesis in an epithelium-autonomous manner, but also suggest that BRCA1 suppresses EMT in tumor cells by repressing PDGFRβ-PKCα signaling.

PDGFRβ is abundantly expressed in normal stromal fibroblasts and in late-stage breast cancer cells, whereas PDGFs, ligands to PDGFRβ, are mainly expressed and secreted in epithelial and carcinoma cells [35–37, 40]. Importantly, in human breast cancers, high stromal PDGFRβ expression is significantly associated with high histopathological grade, ER negativity, and shorter recurrence-free survival [66]. Indeed, some studies suggest that tumor cells secret PDGF-B as a means of recruiting/activating fibroblasts [67, 68]. However, it has not yet been shown if stromal cells provide the PDGFs or what impact stromal cells may have on tumor cells with high PDGFRβ. Our study suggests breast cancer phenotypes attributed to PDGFRβ signaling can be cancer cell intrinsic. Consistent with this, some evidence suggests that breast cancer cells utilize this pathway in an autocrine fashion, producing PDGF-A/B to self-activate PDGFRβ [40].

While studies have not examined the prognostic ramifications of tumor-cell PDGF/PDGFRβ expression, our results here using mouse and human tumors indicate a high likelihood that tumor cell PDGFRβ staining might provide prognostic information in BRCA1/INK4-RB-deficient tumors. In particular, given our results indicating that Pdgfrβ-Pkcα signaling induces molecular indicators of EMT, we anticipate that BRCA1/INK4-RB-deficient tumors might be more invasive and more likely to develop resistance to chemotherapy. Thus, we anticipate these TNBC patients may require targeted therapies individualized to the unique molecular pathways that enhance the malignancy of BRCA1/INK4-RB-deficient tumors.

Target-specific drugs are available for treating HER2-positive cancers and ER-positive luminal type cancers. Very few therapeutic options are available for highly aggressive and metastatic BLBCs. More than half of BLBCs have a dysfunctional BRCA1 pathway and harbor defects in DNA damage repair [21], which make these patients initially respond well to DNA-damaging agents such as cisplatin and PARP inhibitors. However, tumor recurrence and acquired resistance to DNA-damaging agents combine to decrease the 5-year survival of such patients [24, 69]. In this report, we find preclinical evidence that Pdgfrβ-Pkcα might serve as one targetable pathway. In particular, given that pharmaceutical inhibition of PDGFRβ efficiently promotes cell death of BRCA1-deficient tumor cells, it indicates the potential to tailor specific therapies to BLBC patients with BRCA1 deficiency. Thus, as a whole, this study uncovers a targetable PDGFRβ-PKCα pathway with biological and therapeutic importance to TNBC.

## Conclusions

Our work offers the first genetic and biochemical evidence that PDGFRβ-PKCα signaling is repressed by BRCA1, which establishes PDGFRβ-PKCα signaling as a therapeutic target for *BRCA1*-deficient breast cancers. This study not only reveals the molecular mechanism of BRCA1 in suppressing EMT but also tests the efficacy of inhibitors that target PDGFRβ-PKCα signaling on suppressing BRCA1-deficient tumor initiation and progression.

## Supplementary Information

**Additional file 1.** Primer sequence for accessing the occupancy of BRCA1 on the PDGFRβ locus.

**Additional file 2 **The increase of Pdgfrβ in *p18*^*-/-*^*;Brca1*^*+/-*^ mammary tumors is associated with the activated Pdgf signaling as well as EMT and stem cell signatures. (A, B) *p18*^*-/-*^*;Brca1*^*+/-*^ tumors expressing high level of Pdgfrβ (*n* = 9) and low level of Pdgfrβ (*n* = 2) were analyzed for enrichment of Pdgf pathway (A) and of top correlates made up of EMT and stem cell signatures (B). (C, D) Correlation analysis of Pdgfrβ with Zeb1 (C) and Twist (D) in *p18*^*-/-*^*;Brca1*^*+/-*^ tumors.

**Additional file 3 **Brca1-deficient mouse mammary tumors express high level of Pdgfrβ and are metastatic. (A) Representative immunostaining of a *p16*^*-/-*^*;Brca1*^*MGKO*^ mammary tumor and its lung metastasis (M) with antibodies against Pdgfrβ (Green) and Ck14 (Red). Note the widely expressed Pdgfrβ in primary and metastasized tumors that are Ck14 positive. (B) Representative immunostaining of an additional *p18*^*-/-*^*;Brca1*^*MGKO*^ mammary tumor.

**Additional file 4 **Loss of Brca1 activates Pdgfrβ-Pkcα signaling and EMT in mammary tumors. Mammary tumors spontaneously developed in *p18*^*-/-*^ and *p18*^*-/-*^;*Brca1*^MGKO^ mice were analyzed by western blot.

**Additional file 5.** BRCA1 represses transcription of PDGFRβ and EMT-associated genes. SUM149 cells were transfected with pBabe-empty (Empty), pBabe-HA-BRCA1 (BRCA1-a), or pBabe-Myc-BRCA1 (BRCA1-b). Expression of genes indicated in the cells were determined by western blot (A) and qRT-PCR (B) 48 h after transfection.

**Additional file 6 **Pharmaceutical inhibition of PDGFRβ or PKCα activity targets BRCA1 deficient human breast cancer cells. (A, B) HCC1937 (A) and T47D (B) cells treated with DMSO, PDGFR Inh III at 20 nM, or Ro-31-8220 at 35 nM for 24 h were analyzed by western blot. Due to the extremely low level of PDGFRβ and PKCα in T47D cells in comparison with that in HCC1937 cells (shown in Fig. 7b), longer exposure bands for T47D cells were shown in (B). N.S., non-specific band. (C) HCC1937 and T47D cells were treated with DMSO, PDGFR Inh III, or Ro-31-8220 at the indicated concentrations for 24 h, and the number of viable cells was determined. Data are represented as mean ± SD of triplicates. *p < 0.05 between DMSO and drug treated groups by student t test. **p < 0.01 between DMSO and drug treated groups.

**Additional file 7.** Expression of BRCA1 is inversely related with that of PDGFRβ and PKCα in human breast cancers. (A) Representative immunostaining analysis for serial human breast cancer sections. Antibodies used were indicated. (B) Immunostaining results for individual tumor in (A). +/−, < 2%; +, 2–10%; ++, 10–40%; +++, 40–70%; ++++, > 70%.

## Data Availability

All data generated or analyzed during this study are included in this published article and its supplementary information files.

## Abbreviations

ER: Estrogen receptor
EMT: Epithelial-to-mesenchymal transition
p16: p16^INK4A^
p18: p18^INK4C^
BLBCs: Basal-like breast cancers
TNBC: Triple-negative breast cancer
TICs: Tumor-initiating cells
PARP: Poly (ADP-ribose) polymerase
INK4: Inhibitors of the CDK4/6
PDGFs: Platelet-derived growth factors
MET: Mesenchymal-to-epithelial transition
CAFs: Cancer-associated fibroblasts
ChIP: Chromatin-immunoprecipitation
PDGFR Inh III: PDGFR tyrosine kinase inhibitor III
MECs: Mammary epithelial cells
*Brca1*^*MGKO*^: *Brca1*^*f/f*^*;MMTV-Cre* or *Brca1*^*f/−*^*;MMTV-Cre*
IHC: Immunohistochemistry
Vim: Vimentin
E-cad: E-cadherin
MFPs: Mammary fat pads
EMT-TFs: EMT-inducing transcription factors
FACS: Fluorescence-activated cell sorting
ssGSEA: Single sample gene set enrichment analysis
FFPE: Formalin-fixed paraffin-embedded
