# Supplementary material for: PDGFRβ is an essential therapeutic target for BRCA1-deficient mammary tumors
Source: Breast Cancer Res. 2021 Jan 21;23:10. doi: 10.1186/s13058-021-01387-x (PMC7819225; doi:10.1186/s13058-021-01387-x)
Supplement: Supplementary file 1 — Additional file 1. Primer sequence for accessing the occupancy of BRCA1 on the PDGFRβ locus. [file 13058_2021_1387_MOESM1_ESM.pdf]

Additional File 1

Primer sequence for accessing the occupancy of BRCA1 on the PDGFRβ locus

Primer 1	FW<br>RV	TCTCTAGCTCTCCAACCTCCT<br>GACTGTCACTCCTCCTCTCAAC	122bp-site -2652 (-2677~-2657)<br>(-2534~-2555)
Primer 2	FW<br>RV	AATTGAAAACAGACGCACGC<br>GCTCACACCACTATGGGCTT	144bp-site -65 (-129~-110)<br>(-6~13)
Primer 3	FW<br>RV	GATGGGGACGCAGAGTTCAG<br>GGGATCTCGGACTGGCTTTAC	109bp-site 16561 (16484~16503)<br>(16578~16598)
Primer 4	FW<br>RV	CTGCGGGAGTTCTCTGGGTT<br>CCGGGTGACAGTTCCAATACC	114bp-site 18382 (18301~18320)<br>(18433~18453)
