# Supplementary figures and images for: PDGFRβ is an essential therapeutic target for BRCA1-deficient mammary tumors
Source: Breast Cancer Res. 2021 Jan 21;23:10. doi: 10.1186/s13058-021-01387-x (PMC7819225; doi:10.1186/s13058-021-01387-x)

A

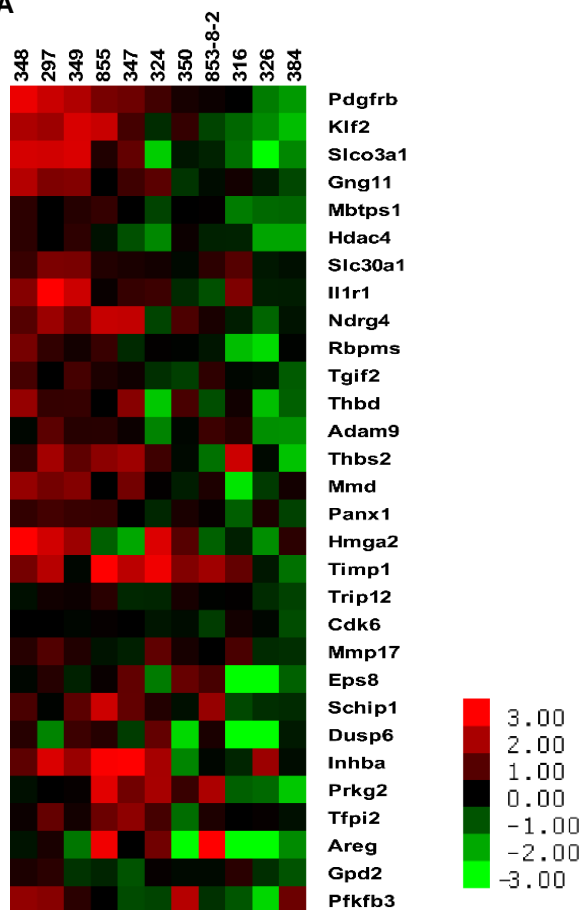

B

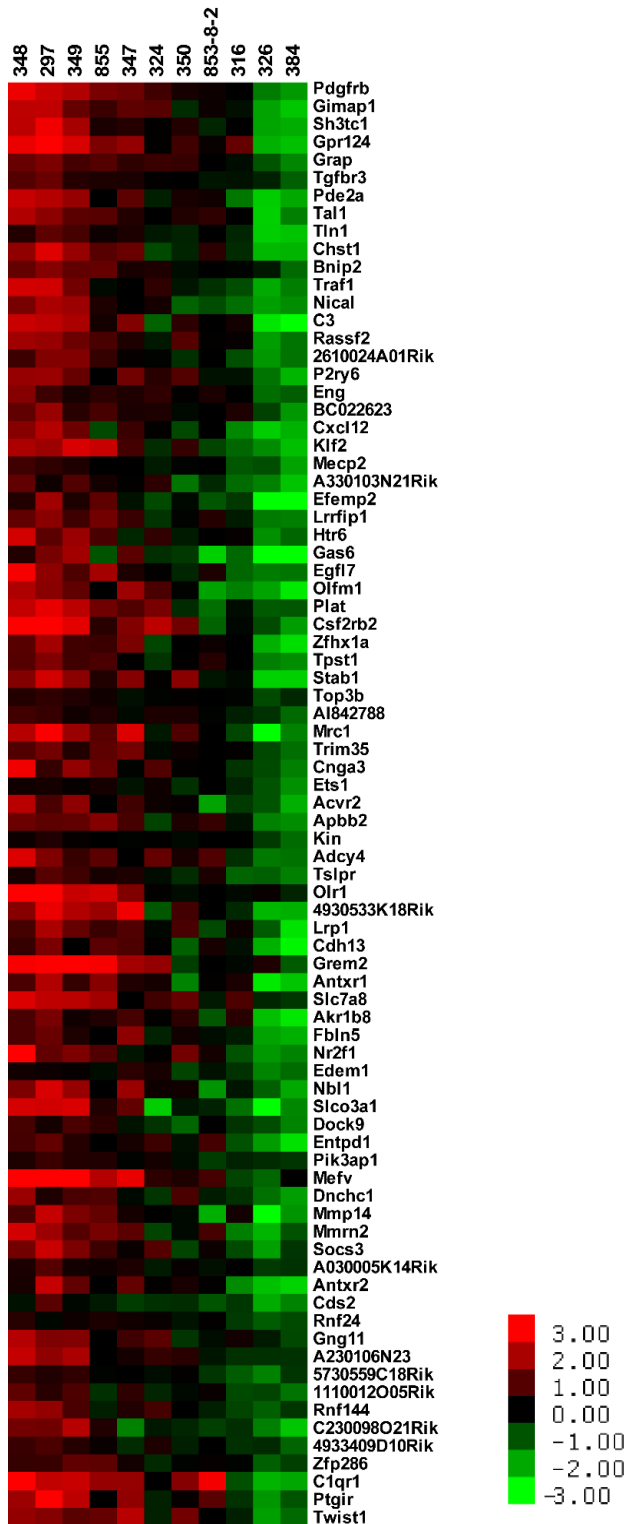

C

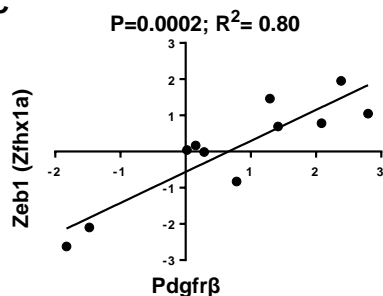

D

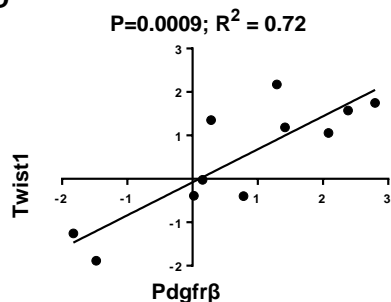

Supplement: Supplementary file 2 — Additional file 2 The increase of Pdgfrβ in p18-/-;Brca1+/- mammary tumors is associated with the activated Pdgf signaling as well as EMT and stem cell signatures. (A, B) p18-/-;Brca1+/- tumors expressing high level of Pdgfrβ (n = 9) and low level of Pdgfrβ (n = 2) were analyzed for enrichment of Pdgf pathway (A) and of top correlates made up of EMT and stem cell signatures (B). (C, D) Correlation analysis of Pdgfrβ with Zeb1 (C) and Twist (D) in p18-/-;Brca1+/- tumors. [file 13058_2021_1387_MOESM2_ESM.pdf]

A

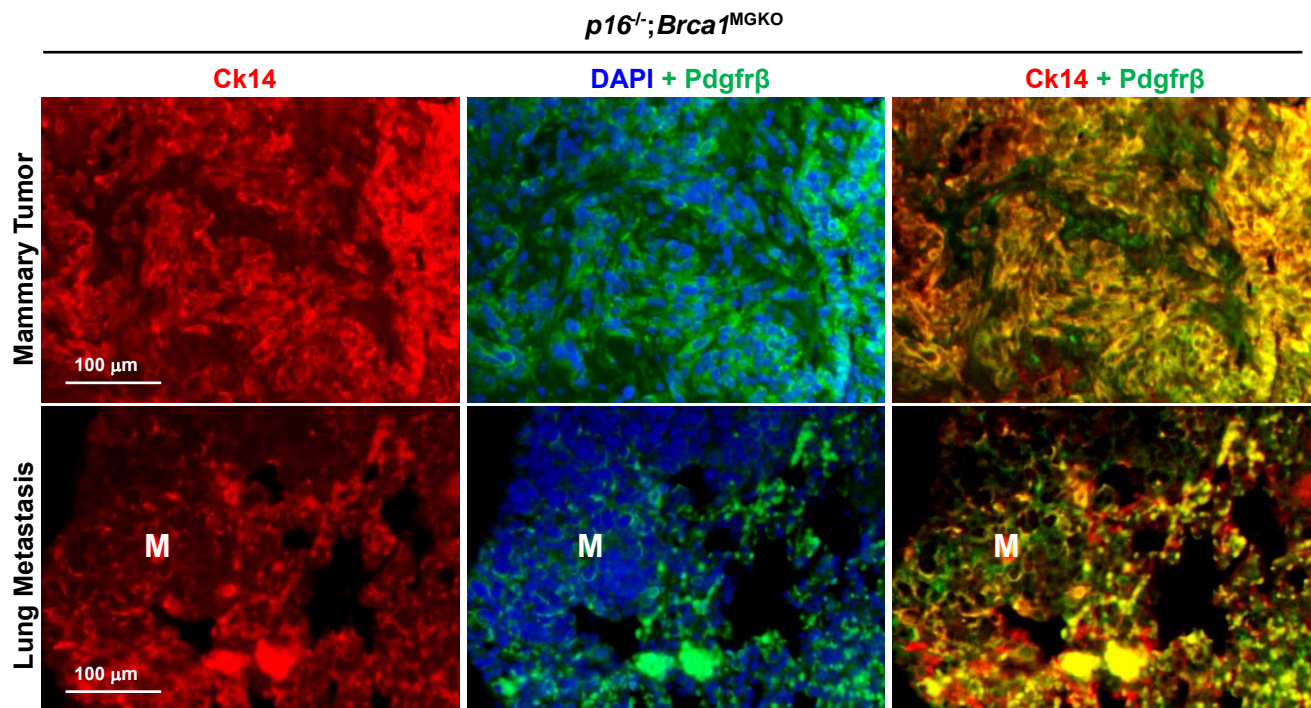

B

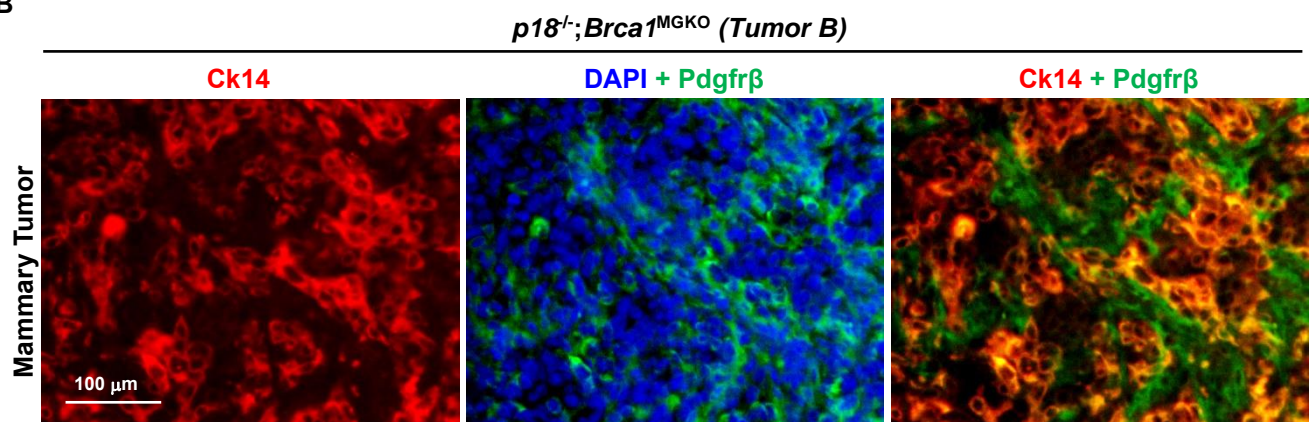

Supplement: Supplementary file 3 — Additional file 3 Brca1-deficient mouse mammary tumors express high level of Pdgfrβ and are metastatic. (A) Representative immunostaining of a p16-/-;Brca1MGKO mammary tumor and its lung metastasis (M) with antibodies against Pdgfrβ (Green) and Ck14 (Red). Note the widely expressed Pdgfrβ in primary and metastasized tumors that are Ck14 positive. (B) Representative immunostaining of an additional p18-/-;Brca1MGKO mammary tumor. [file 13058_2021_1387_MOESM3_ESM.pdf]

Additional File 4

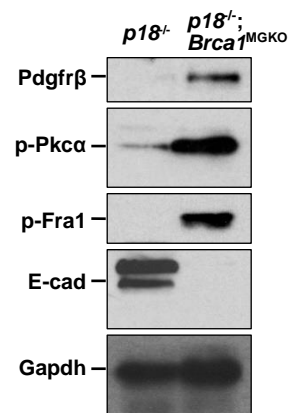

Supplement: Supplementary file 4 — Additional file 4 Loss of Brca1 activates Pdgfrβ-Pkcα signaling and EMT in mammary tumors. Mammary tumors spontaneously developed in p18-/- and p18-/-;Brca1MGKO mice were analyzed by western blot. [file 13058_2021_1387_MOESM4_ESM.pdf]

A

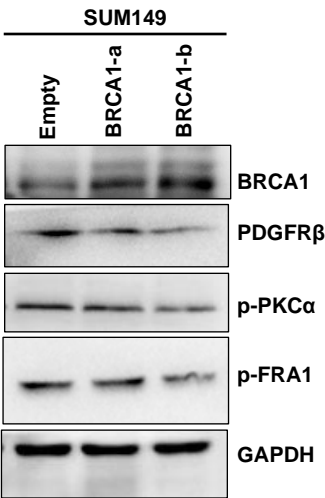

B

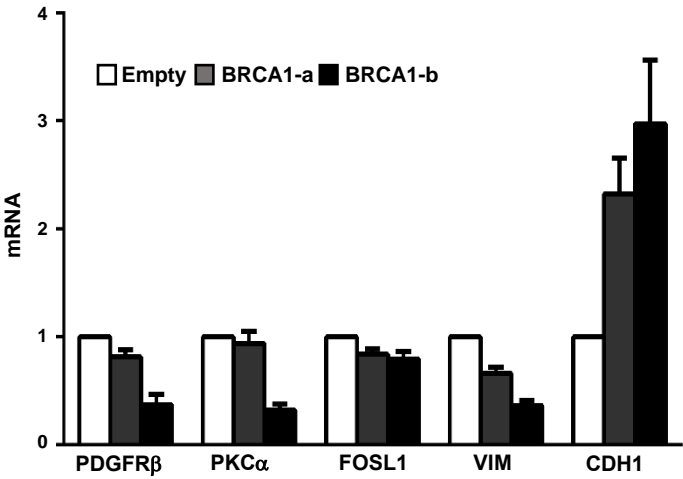

Supplement: Supplementary file 5 — Additional file 5. BRCA1 represses transcription of PDGFRβ and EMT-associated genes. SUM149 cells were transfected with pBabe-empty (Empty), pBabe-HA-BRCA1 (BRCA1-a), or pBabe-Myc-BRCA1 (BRCA1-b). Expression of genes indicated in the cells were determined by western blot (A) and qRT-PCR (B) 48 h after transfection. [file 13058_2021_1387_MOESM5_ESM.pdf]

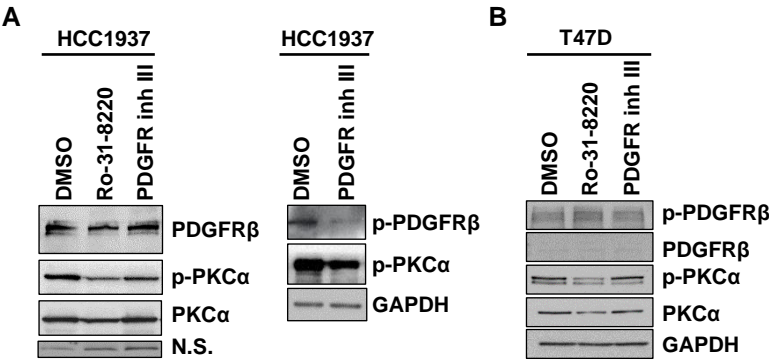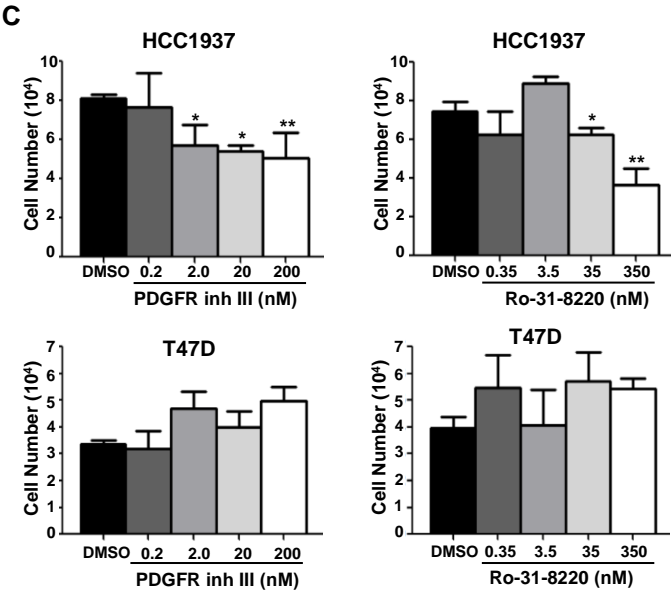

Supplement: Supplementary file 6 — Additional file 6 Pharmaceutical inhibition of PDGFRβ or PKCα activity targets BRCA1 deficient human breast cancer cells. (A, B) HCC1937 (A) and T47D (B) cells treated with DMSO, PDGFR Inh III at 20 nM, or Ro-31-8220 at 35 nM for 24 h were analyzed by western blot. Due to the extremely low level of PDGFRβ and PKCα in T47D cells in comparison with that in HCC1937 cells (shown in Fig. 7b), longer exposure bands for T47D cells were shown in (B). N.S., non-specific band. (C) HCC1937 and T47D cells were treated with DMSO, PDGFR Inh III, or Ro-31-8220 at the indicated concentrations for 24 h, and the number of viable cells was determined. Data are represented as mean ± SD of triplicates. *p < 0.05 between DMSO and drug treated groups by student t test. **p < 0.01 between DMSO and drug treated groups. [file 13058_2021_1387_MOESM6_ESM.pdf]

A

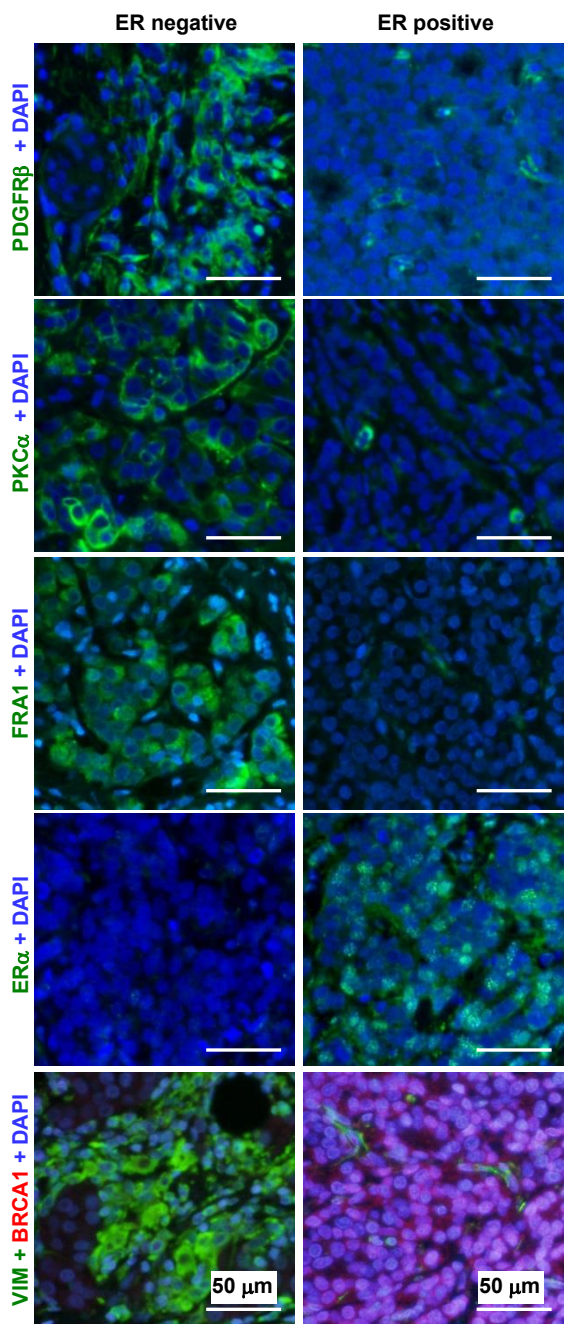

B

| #  | ER | BRCA1 | PDGFRβ | PKCα | FRA1 | VIM |
|----|----|-------|--------|------|------|-----|
| 1  | +  | ++++  | -      | -    | -    | -   |
| 2  | +  | +++   | -      | -    | -    | -   |
| 3  | +  | +++   | -      | -    | -    | -   |
| 4  | +  | ++    | -      | -    | -    | -   |
| 5  | +  | ++    | -      | +/-  | +/-  | +/- |
| 6  | +  | ++    | +/-    | +/-  | +    | +/- |
| 7  | +  | +     | +      | +/-  | +/-  | +   |
| 8  | +  | +     | -      | +/-  | +/-  | +   |
| 9  | -  | +/-   | +/-    | +    | +    | +   |
| 10 | -  | +/-   | +      | +    | +    | +/- |
| 11 | -  | -     | -      | +/-  | +/-  | +   |
| 12 | -  | -     | +/-    | +    | +/-  | +   |
| 13 | -  | -     | +      | +    | +    | +   |
| 14 | -  | -     | +      | ++   | +    | +   |
| 15 | -  | -     | +      | ++   | ++   | ++  |
| 16 | -  | -     | ++     | ++   | +++  | ++  |
| 17 | -  | -     | ++     | ++   | +++  | +++ |
| 18 | -  | -     | ++     | +++  | +++  | ++  |

Supplement: Supplementary file 7 — Additional file 7. Expression of BRCA1 is inversely related with that of PDGFRβ and PKCα in human breast cancers. (A) Representative immunostaining analysis for serial human breast cancer sections. Antibodies used were indicated. (B) Immunostaining results for individual tumor in (A). +/−, < 2%; +, 2–10%; ++, 10–40%; +++, 40–70%; ++++, > 70%. [file 13058_2021_1387_MOESM7_ESM.pdf]
